# Functional Studies of β-Glucosidases of *Cytophaga hutchinsonii* and Their Effects on Cellulose Degradation

**DOI:** 10.3389/fmicb.2017.00140

**Published:** 2017-02-02

**Authors:** Xinfeng Bai, Xifeng Wang, Sen Wang, Xiaofei Ji, Zhiwei Guan, Weican Zhang, Xuemei Lu

**Affiliations:** ^1^State Key Laboratory of Microbial Technology, School of Life Science, Shandong UniversityJinan, China; ^2^Department of Pathogenic Biology, Binzhou Medical UniversityYantai, China

**Keywords:** *Cytophaga hutchinsonii*, β-glucosidase, cellobiose, cellulose, degradation

## Abstract

*Cytophaga hutchinsonii* can rapidly digest crystalline cellulose without free cellulases or cellulosomes. Its cell-contact cellulose degradation mechanism is unknown. In this study, the four β-glucosidase (bgl) genes in *C. hutchinsonii* were singly and multiply deleted, and the functions of these β-glucosidases in cellobiose and cellulose degradation were investigated. We found that the constitutively expressed BglB played a key role in cellobiose utilization, while BglA which was induced by cellobiose could partially make up for the deletion of *bglB*. The double deletion mutant Δ*bglA*/*bglB* lost the ability to digest cellobiose and could not thrive in cellulose medium, indicating that β-glucosidases were important for cellulose degradation. When cultured in cellulose medium, a small amount of glucose accumulated in the medium in the initial stage of growth for the wild type, while almost no glucose accumulated for Δ*bglA*/*bglB*. When supplemented with a small amount of glucose, Δ*bglA*/*bglB* started to degrade cellulose and grew in cellulose medium. We inferred that glucose might be essential for initiating cellulose degradation, and with additional glucose, *C. hutchinsonii* could partially utilize cellulose without β-glucosidases. We also found that there were both cellulose binding cells and free cells when cultured in cellulose. Since direct contact between *C. hutchinsonii* cells and cellulose is necessary for cellulose degradation, we deduced that the free cells which were convenient to explore new territory in the environment might be fed by the adherent cells which could produce cello-oligosaccharide and glucose into the environment. This study enriched our knowledge of the cellulolytic pathway of *C. hutchinsonii*.

## Introduction

Cellulose is the most abundant biopolymer on earth (Bayer and Lamed, 1992). The β-1,4-linked glucose chains of cellulose form highly ordered crystalline fibrils that are relatively recalcitrant to degradation (Falkowski et al., 2000; Wilson, 2008). Cellulolytic microorganisms use different strategies to degrade cellulose (Wilson, 2008). Most aerobic cellulolytic bacteria and fungi secret a set of individual cellulases, which act synergistically to degrade cellulose into cello-oligosaccharides (mainly cellobiose), and these cello-oligosaccharides are then degraded into glucose by β-glucosidase (Wilson, 2008; Kovacs et al., 2009). Many anaerobic microorganisms use cellulosomes, large multienzyme complexes, to degrade cellulose into cello-oligosaccharide on the cell surface (Beguin and Lemaire, 1996; Bayer et al., 1998; Zhang and Lynd, 2005). Then the cello-oligosaccharide is absorbed into the cell by ATP-binding cassette transporters (Strobel et al., 1995; Nataf et al., 2009) and cleaved via either phosphorolytic or hydrolytic reactions by phosphorylases and β-glucosidases, respectively (Ng and Zeikus, 1986; Zhang and Lynd, 2004).

*Cytophaga hutchinsonii* is a widely distributed Gram-negative cellulolytic bacterium (Walker and Warren, 1938; Stanier, 1942; Xie et al., 2007). Direct contact between *C. hutchinsonii* cells and insoluble cellulose is necessary for cellulose degradation (Walker and Warren, 1938; Stanier, 1942), and most of the cellulase activity appears to be cell associated (Chang and Thayer, 1977; Xie et al., 2007). *C. hutchinsonii* uses a novel strategy to degrade crystalline cellulose without free cellulase and cellulosomes. Though *C. hutchinsonii* has been studied for many years, the mechanism for its cello-oligosaccharide and cellulose utilization is still unknown. Since no apparent cellulose degradation products were detected in the medium (Chang and Thayer, 1977), Wilson speculated that individual cellulose molecules were removed from cellulose fibers and transported into the periplasmic space, where they could be digested by cellulases (Wilson, 2009a). Analysis of the genomic sequence revealed that the cellulolytic system of *C. hutchinsonii* consists of nine potential endo-glucanases and four β-glucosidases. However, it lacks exo-glucanases which are considered to play important roles in crystalline cellulose digestion, and most of the proteins related to endo-glucanases do not contain recognizable CBMs to bind insoluble cellulose (Xie et al., 2007; Wilson, 2008, 2009b). Recently, Zhu studied the function of these endo-glucanases and reported that the periplasmic endo-glucanases played crucial roles in cellulose degradation (Zhu et al., 2016). Cellobiose is the primary unit of cellulose and an important intermediate product of cellulose degradation. Cellobiose can influence the expression of cellulases and the degradation of cellulose (Freier et al., 1988; Yoshida et al., 2004; Xu et al., 2014). However, the mechanisms of cellobiose and other cello-oligosaccharides utilization by *C. hutchinsonii* have not been studied and the effects of β-glucosidases on cellulose utilization are still unknown.

In this study, all of the four putative β-glucosidases were singly and multiply deleted. The cellobiose and cellulose utilization abilities of the mutants were studied and the cellulose degradation products in the supernatant and in cells were both investigated by ion chromatography. The function of these β-glucosidases in cello-oligosaccharides and cellulose utilization was studied.

## Materials and Methods

### Bacterial Strains, Plasmids, and Growth Conditions

*Cytophaga hutchinsonii* ATCC 33406 was kindly provided by Mark J. McBride and grown at 30°C in Stanier medium (Stanier, 1942). *Escherichia coli* strains were grown in Luria-Bertani medium at 37°C. Bacterial strains and plasmids used in this study are listed in **Table 1**. Primers are listed in **Supplementary Table S1**. To test the utilization of different carbohydrate sources, *C. hutchinsonii* was grown in Stanier medium supplemented with 0.2% (wt/vol) glucose, 0.2% (wt/vol) cellobiose, or 0.4% (wt/vol) Avicel cellulose (PH-101, Omega, GA, USA) as the sole carbon source. To analyze cellulase activity, cells were grown in Stanier medium supplemented with 0.2% (wt/vol) glucose or 0.4% (wt/vol) Avicel at 30°C. Antibiotics were used at the following concentrations: ampicillin (Ap), 100 μg/mL; erythromycin (Em), 30 μg/mL; cefoxitin (Cfx), 15 μg/mL; and chloramphenicol (Cm), 15 μg/mL.

**Table 1:** Strains and plasmids used in this study.

Strain or plasmid	Description^a^	Reference
***E. coli* strains**		
DH5α	Strain used for gene cloning	Clontech
***C. hutchinsonii* strains**		
ATCC 33406	Wild type	ATCC
Δ*bglA* strain	Deletion of *bglA* (Em^r^)	This study
Δ*bglB* strain	Deletion of *bglB* (Em^r^)	This study
Δ*bglC* strain	Deletion of *bglC* (Em^r^)	This study
Δ*bglD* strain	Deletion of *bglD* (Em^r^)	This study
Δ*bglA*/*bglB* strain	Double deletion of *bglA* and *bglB* (EM^r^ CM^r^)	This study
Δ*bglA*/*bglB/bglC* strain	Triple deletion of *bglA*, *bglB* and *bglC* (EM^r^ CM^r^ CfX^r^)	This study
Δ*bglA*/*bglB::*pCFX*bglA* strain	Complementation of Δ*bglA*/*bglB* with pCFX*bglA*	This study
Δ*bglA*/*bglB::*pCFX*bglA*^∗^ strain	Complementation of Δ*bglA*/*bglB* with pCFX*bglA*^∗^	This study
Δ*bglA*/*bglB::*pCFX*bglB* strain	Complementation of Δ*bglA*/*bglB* with pCFX*bglB*	This study
Δ*bglA*/*bglB::*pCFX*bglB*^∗^ strain	Complementation of Δ*bglA*/*bglB* with pCFX*bglB*^∗^	This study
**Plasmids**		
pSJHS	Gene-targeting template plasmid carrying *ermF*; Ap^r^ (Em^r^)	Wang et al., 2014
pSJHC	Gene-targeting template plasmid carrying *cat* under the control of the *ompA* promoter from *F. johnsoniae*; Ap^r^ (Cm^r^)	Wang et al., 2014
pSJHCFX	Similar to pSJHC except for carrying *cfxA* instead of *cat*; Ap^r^ (CfX^r^)	This study
pCH	Gene complementation plasmid carrying *cat*; Ap^r^ (Cm^r^)	Ji et al., 2014
pCFX	Similar to pSJHC except for carrying *cfxA* instead of *cat*; Ap^r^ (Cfx^r^)	This study
pCFX*bglA*	A 2.4-kbp fragment spanning *bglA* amplified with primers C*bglA*-H1F and C*bglA*-H2R and ligated into *Sac*I and *Sal*I sites of pCFX; oriC; Ap^r^ (Cfx^r^)	This study
pCFX*bglA*^∗^	Similar to pCF*bglA* except that the residue of D308 of BglA was changed into Ala; oriC; Ap^r^ (Cfx^r^)	This study
pCFX*bglB*	A 2.8-kbp fragment spanning *bglB* amplified with primers C*bglB*-H1F and C*bglB*-H2R and ligated into *Sac*I and *Sal*I sites of pCFX; oriC; Ap^r^ (Cfx^r^)	This study
pCFX*bglB*^∗^	Similar to pCFX*bglB* except that the residue of D321 of BglB was changed into Ala; oriC; Ap^r^ (Cfx^r^)	This study


*^a^Antibiotic resistance phenotypes: Ap^r^, ampicillin; Cm^r^, chloramphenicol; Em^r^, erythromycin; Cf^r^, cefoxitin; Km^r^, kanamycin. Phenotypes in parentheses are expressed in *C. hutchinsonii*, and phenotypes not in parentheses are expressed in *E. coli*. Asterisk was used to mark the site mutational complemented plasmids and strains.*

### Quantitative Reverse Transcription-PCR (RT-PCR)

Cells were cultured to middle exponential phase in Stanier medium with 0.2% (wt/vol) glucose or 0.2% (wt/vol) Avicel as carbon source. Total RNA was isolated by a bacterial RNA kit (Omega, Norcross, GA, USA). Elimination of traces of DNA was carried out with the genomic DNA (gDNA) Eraser from TaKaRa (Dalian, China) according to the manufacturer’s instructions. Quantitative PCRs were performed using a Bio-Rad myIQ2 thermocycler (Bio-Rad) and the SYBR green supermix (TaKaRa). Data analysis was performed using the 2[-delta delta C (T)] method and were normalized to an endogenous control (16S rDNA) with expression as the reference. Three biological repeats were set for all assays. Prime pairs 16*SrRNA*-1/16*SrRNA*-2, RT-*bglA*-1/RT-*bglA*-2, RT-*bglB*-1/RT-*bglB*-2, RT-*bglC*-1/RT-*bglC*-2, and RT-*bglD*-1/RT-*bglD*-2 were used to amplify *bglA*, *bglB*, *bglC*, and *bglD*, respectively. Primers are listed in **Supplementary Table S1**.

### Localization of the β-Glucosidases

*Cytophaga hutchinsonii* strains were grown in Stanier medium supplemented with 0.2% (wt/vol) glucose at 30°C to middle exponential phase. Cells were collected through centrifugation at 5,000 × *g* for 10 min. Then the cells were resuspended in fresh Stanier medium supplemented with 0.2% (wt/vol) cellobiose and 0.2% (wt/vol) glucose, and induced for 4 h. The total membrane proteins and the soluble proteins were prepared as described by Zhou et al. (2015). Briefly, *C. hutchinsonii* cells were collected at 5,000 × *g* and 4°C for 10 min. The pellet was washed with piperazine-1,4-bis (2-ethanesulfonic acid) (PIPES) buffer (50 mM, pH 6.8), disrupted by sonication, and cell debris was removed by centrifugation (15,000 × *g*, 20 min). Cell lysates were subjected to ultra-centrifugation (Beckman, Fullerton, CA, USA) at 100,000 × *g* for 1 h at 4°C. The supernatant was collected as soluble protein fractions. The membrane proteins were solubilized from pellets by PIPES buffer (pH 6.8) including 2 % (v/v) TritonX-100 at 4°C over night. Then the suspension was again ultracentrifuged at 100,000 × *g* for 30 min at 4°C to obtain the supernatant as total membrane protein fractions. The outer membrane protein preparation was performed as described by Ji et al. (2014). Briefly, *C. hutchinsonii* cells were collected at 5,000 × *g* and 4°C for 10 min. The pelleted cells were washed with 50 mM PIPES buffer (pH 6.8), resuspended in PIPES buffer with 0.5 M NaCl, and then incubated at 4°C for 15 min with shaking at 150 rpm. Cells were removed by centrifugation at 12,000 × *g* for 20 min at 4°C, and the supernatant containing the buffer-washed proteins was ultracentrifuged at 100,000 × *g* for 30 min at 4°C. The sediment was resuspended in PIPES buffer as outer membrane proteins. 1 mM phenylmethylsulfonyl fluoride (PMSF) was added to deactivate proteases.

The renatured SDS-PAGE of the above proteins was carried out as described by Kwon et al. (1994). Briefly, samples were neither boiled nor treated with β-mercaptoethanol. Electrophoresis was performed using an 8 cm × 10 cm, 10% polyacrylamide gel at a current of 20 mA for 2 h. The concentration of the SDS in the loading buffer, running buffer and the gel were the same as that of the ordinary SDS-PAGE. Subsequently, the gel was placed in a 0.05% (vol/vol) Triton X-100 solution for 30 min at 4°C to eliminate SDS, then the gel was placed in a 0.1 M citric-Na_2_HPO_4_ buffer (pH 6.8) with 0.1% (wt/vol) esculin and 0.05% (wt/vol) ferric chloride at 30°C for 30 min. During incubation, black bands corresponding to the β-glucosidases appeared against a transparent background. The bands were excised from the gel and the proteins were identified by MALDI-TOF mass spectrometry.

For Western blot analysis, proteins in the SDS-PAGE gel were transferred onto 0.45-μm-pore-size PVDF membranes (Immobilon-P; Millipore, MA, USA) using a semidry electrophoretic transfer cell (Bio-Rad, Hercules, CA, USA), according to the manufacturer’s instructions. Membranes were blocked with skim milk and probed with anti-BglA and anti-BglB rabbit antiserums. Anti-BglA was raised to the 678-amino-acid region of BglA (from Lys^79^ to Asn^757^). Anti-BglB was raised to the 800-amino-acid region of BglB (from Cys^20^ to Glu^819^). After incubation with horseradish peroxidase (HRP)-conjugated goat anti-rabbit IgG (Cowin Biotech, Beijing, China) as a secondary antibody, proteins were detected by the chemiluminescent HRP substrate (Immobilon Western, Millipore, MA, USA) according to the manufacturer’s instructions, and the film was processed by an automatic X-ray film processor (SMPIC 2600C-1; Shanghai, China).

### Construction of the β-Glucosidase Deletion Mutants

The four predicted β-glucosidase genes were deleted, respectively, by a double-crossover recombination system as described by Wang et al. (2014). Briefly, the 2-kb fragments of the upstream and downstream of β-glucosidase genes were amplified from *C. hutchinsonii* genomic DNA and successively ligated into the pSJHS plasmid to yield the disruption vector. The gene-targeting cassette was amplified by PCR and purified with a Cycle Pure kit (Omega, GA, USA). A total of 1.5 μg of PCR product was transformed into 100 μL of competent cells of *C. hutchinsonii* by electroporation and grown on PY6 agar (6 g of peptone, 0.5 g of yeast extract, 4 g of glucose,10 g of agar per liter, pH 7.3) containing erythromycin at 30°C (Wang et al., 2014). The pSJHC (Cm^r^) and pSJHCFX (Cfx^r^) plasmids were used to construct the disruption vectors for multiple gene deletion (Ji et al., 2014).

### Measurement of Cellulase Activity

Cells were grown in Stainer medium supplemented with 0.2% (wt/vol) glucose or 0.4% (wt/vol) Avicel. Cells of the middle exponential phase were gathered through centrifugation at 5,000 × *g* for 10 min. For intact cell samples, cell pellets were washed with Na_2_HPO_4_-KH_2_PO_4_ buffer (50 mM, pH 6.8) and resuspended in the same buffer. For cell extract samples, cell pellets were washed and resuspended with Na_2_HPO_4_-KH_2_PO_4_ buffer containing 2% (vol/vol) Triton X-100. Then the mixture was incubated at 4°C for 4 h to make sure that all the proteins were released into the buffer. Sodium carboxymethyl cellulose (CMC-Na) and *p*-nitrophenyl β-D-glucopyranoside (*p*NPG) were purchased from Sigma-Aldrich (St. Louis, MO, USA) and used as substrates to measure endo-glucanase and β-glucosidase activities, respectively, according to previously described methods (Ji et al., 2014). Protein concentrations were quantified as described by Bradford (1976), and all the enzymatic assays were carried out in triplicate.

### Measurement of Growth Property in Liquid Culture

To detect the growth property of the wild type strain and the β-glucosidase deletion mutants, the growth curses were measured by a Bioscreen C analyzer (Oy growth curves Ab Ltd, Finland). All the strains were grown in Stanier medium supplemented with 0.2% (wt/vol) glucose to middle exponential phase and then 3% (vol/vol) cells were inoculated into 200 μL of Stanier medium supplemented with 0.2% (wt/vol) glucose or 0.2% (wt/vol) cellobiose as the sole carbon source in a sample plate. The plate was incubated at 30°C with medium speed shaking, and the growth was monitored by the optical density at 600 nm. When Avicel was used as the carbon source, incubations were done in 300 mL flasks with shaking (160 rpm) at 30°C. To measure the growth of *C. hutchinsonii*, total cellular protein was quantified as described by Bradford (1976). The weight of residual Avicel was measured as described by Zhu et al. (2010).

### Cellulose Degradation Assay

Cellulose degradation assays were carried out as described by Ji et al. (2012). Equivalent amounts of cells from PY6 medium were spotted on Whatman number 1 filter paper, which was preplaced on the top of solid Stanier medium (Wang et al., 2014) with 10 g/liter agar or 15 g/liter phytagel (Sigma-Aldrich, USA), and the plates were incubated at 30°C to observe cellulose degradation.

### Preparation of the Extracellular Degradation Products

To detect the degradation products in the medium, middle exponential phase cells, which were grown in glucose medium, were washed with Stanier medium and incubated with 0.2% (wt/vol) cellobiose, 0.4% (wt/vol) Avicel or 0.2% (wt/vol) cello-oligosaccharide mixture, respectively. Samples were incubated at 30°C with shaking at 160 rpm. After incubation, the supernatant which contained the extracellular degradation products was collected through centrifugation at 10,000 × *g* for 10 min to remove the cells, and subsequently filtered through a 0.22 μm-pore-size polyvinylidene difluoride (PVDF) filter (Sangon, Shanghai, China). The cello-oligosaccharide mixture, which is the acidolysis product of cellulose, was prepared as described by Zhang and Lynd (2003).

### Extraction of the Cello-oligosaccharides in the Cell

Cells were collected through centrifugation at 5,000 × *g* for 10 min, and the cell pellets were washed three times with Stanier medium. The Stanier medium after washing the cells for the third time was taken as a control sample to make sure all the metabolites in the medium had been removed. Then cell pellets were resuspended in deionized water and disrupted by sonication. The supernatant which contained the intracellular cello-oligosaccharides was centrifuged at 100,000 × *g* for 30 min and subsequently filtered through a 0.22 μm-pore-size PVDF filter to remove residue. Proteins were measured to quantify the cell concentration, and the samples extracted from the same amount of cells were detected by ion chromatography.

When *C. hutchinsonii* was cultured in cellulose medium, large amounts of the cells were absorbed on the cellulose but there were also some free cells suspended in the medium. To separate the free cells and the cellulose-bound cells, cells and the residual cellulose were first collected through centrifugation at 5,000 × *g* for 10 min. Then the cell and cellulose mixture was resuspended in Stanier medium, followed by centrifugation at 100 × *g* for 5 min. The supernatant containing the free cells was transferred to a new tube. This was done three times to make sure all the free cells were separated from the mixture. Subsequently, the free cells and cellulose-bound cells were collected through centrifugation at 5,000 × *g* for 10 min. The free cells and cellulose-bound cells were washed with Stanier medium as described above and then disrupted by sonication. The supernatants which contained intracellular cello-oligosaccharides were extracted as described above.

### Detection of the Cello-oligosaccharides

Ion chromatography with integrated pulsed amperometric detection (IC-IPAD) (Thermo Scientific Dionex ICS-5000+, USA) was used to detect the degradation products of cellulose and cello-oligosaccharide. Ion chromatography experimental conditions: flow rate = 1.0 mL/min, injection volume = 25 μL. Eluent conditions were as follows: 100 mM NaOH [isocratic (0.0-1.1 min; inject 1.0 min), a gradient of 0-500 mM NaOAc in 100 mM NaOH (1.1-20.0 min), and return to 100 mM NaOH (20.1-25.0 min) to re-equilibrate the column to the starting conditions prior to injection]. Cello-oligosaccharides were identified by comparing retention times and spikes with purchased standards (G1-G4) and acidolysis products of cellulose. The glucose and cellobiose standard curves with concentration range from 0.1 μg/ml to 0.1 mg/ml were drawn, respectively.

### Complementation of the bgl Deletion Mutants

The replicative plasmid pCFX used for complementation of genes in *C. hutchinsonii* was constructed from plasmid pCH (Ji et al., 2014), in which the chloramphenicol acetyltransferase gene (cat) was replaced by the cefoxitin resistance gene (cfx). A 2.4-kbp fragment spanning *bglA*, 360 bp upstream of the start codon and 150 bp downstream of the stop codon, was amplified with primers C*bglA*-H1F and C*bglA*-H2R. The fragment was digested with *Sac*I and *Sal*I and ligated into the corresponding sites of pCFX to generate pCFX*bglA*. Plasmid pCFX*bglA* was then electroporated into the Δ*bglA*/*bglB* mutant and selected by cefoxitin resistance. Δ*bgla*/*bglb::*pCFX*bglA* refers to complemented strain of the Δ*bglA*/*bglB* mutant with pCFX*bglA*. The molecular structure of BglA and BglB were predicted by the SWISS-MODEL workplace http://swissmodel.expasy.org/ based on the template of the crystal structure of the catalytic domain of *Hordeum vulgare* ExoI (Varghese et al., 1999). The predicted active site residue D308 (BglA) and D321 (BglB) were changed into alanine by overlap extension PCR (Zhang et al., 2014). Then the active site mutational complemented train Δ*bglA*/*bglB::*pCFX*bglA*^∗^ was constructed. Δ*bglA*/*bglB::*pCFX*bglB* and Δ*bglA*/*bglB::*pCFX*bglB*^∗^ were constructed in the same way.

## Results

### The β-Glucosidase Genes of *C. hutchinsonii* and Their Transcription Level in Different Culture Conditions

Analysis of the *C. hutchinsonii* genome showed that *C. hutchinsonii* has four candidate β-glucosidase genes: *bglA* (*chu_2268*, accession number: ABG59531), *bglB* (*chu_2273*, accession number: ABG59535), *bglC* (*chu_3577*, accession number: ABG60810), and *bglD* (*chu_3784*, accession number: ABG61016). All of them belong to glycoside hydrolase family GH3. BglA, BglB, and BglC have previously been predicted to be lipoproteins. There is a signal peptide in BglB, but not in BglA, BglC, and BglD as predicted by SignalP 4.1 (Petersen et al., 2011). However, many hydrophobic amino acids are in the first 30 amino acids of BglA, BglC and BglD, suggesting there might be signal peptides in these proteins.

**FIGURE 1:**
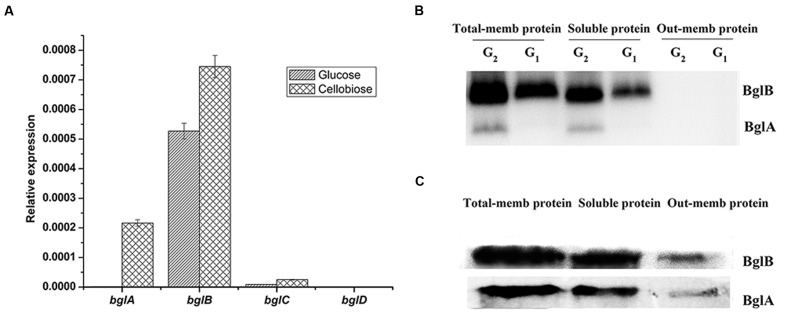
**Expression and distribution pattern of the β-glucosidases.** **(A)** Quantitative RT-PCR analysis of the expression of β-glucosidases in the wild type strain of *C. hutchinsonii* in glucose culture and cellulose. Expression levels of 16srRNA were used as an endogenous control in all samples and expression levels of 16srRNA were normalized to 1. Values are the mean of three biological replicates. Error bars are the standard deviations from these replicates. **(B)** β-Glucosidases renatured SDS-PAGE assay of total membrane protein (total-mem protein), soluble protein and outer membrane protein (outer-mem protein). Proteins were extracted from glucose culture (G_1_) and cellobiose culture (G_2_). **(C)** Western blot analysis of BglA and BglB in different protein components. Proteins were extracted from wild type cells induced by 0.4% (wt/vol) cellobiose for 4 h.

In order to detect the transcription level of the β-glucosidases at mRNA level, we used the quantitative RT-PCR assay to detect these genes both in glucose culture and cellulose culture. As shown in **Figure 1A**, *bglB* was the main β-glucosidase gene transcribed in glucose culture. *bglA* was not transcribed in glucose culture but could be induced by cellulose. *bglA* and *bglB* were the two main β-glucosidase genes transcribed in cellulose culture. The transcription level of *bglC* was very low both in glucose and cellulose condition. And the transcription of *bglD* was undetectable under our experimental condition. In addition, the transcription patterns of the four β-glucosidase genes in cellobiose culture were similar to those in cellulose culture (data not shown).

### Expression and Distribution of β-Glucosidases

The expression and distribution of the β-glucosidases were detected. The total-membrane protein, soluble protein and outer membrane protein of the wild type strain were extracted as described in Section “Materials and Methods.” A renatured SDS-PAGE was performed as described in the Section “Materials and Method,” and β-glucosidases were detected by dyeing with esculin and ferric chloride. As shown in **Figure 1B**, there were two active bands identified as BglB and BglA by MALDI-TOF mass spectrometry. No active band of BglC or BglD was detected in our experiment. This result showed that BglB constitutively expressed both in glucose and cellobiose cultures, while BglA could only be detected in cellobiose culture, which was consistent with the result of the quantitative RT-PCR. In addition, the brightness (reflects the activity of the β-glucosidases) of the BglB band was higher than that of BglA. As shown in **Figure 1B**, BglA and BglB mainly presented in the membrane sample and some in the soluble sample. No active band was detected in the outer-membrane protein sample of the renatured PAGE (**Figure 1B**), and only little amount of BglA and BglB could be detected in the outer-membrane protein of the Western blot assay (**Figure 1C**). This might have been caused by the high sensitivity of the Western blot assay and the residual amounts of β-glucosidases in the outer membrane fraction of the Western blot assay might be caused by the contamination of periplasmic proteins. BglA and BglB were previously predicted to be lipoproteins (Xie et al., 2007). In bacteria, lipoproteins are mainly attached to either the cytoplasmic or outer membrane by lipid moiety (Ichihara et al., 1981; Wu et al., 1982). So we predicted that the β-glucosidases in *C. hutchinsonii* were mainly located in the periplasmic space.

### Construction of the β-Glucosidase Deletion Mutants

In order to analyze the function of all the β-glucosidases in *C. hutchinsonii*, the four β-glucosidases were singly deleted (**Supplementary Figure S1**) by the double-crossover recombination system as described in the Section “Materials and Methods.” Then the two main active β-glucosidases (*bglA* and *bglB*) and the three expressed β-glucosidases (*bglA*, *bglB*, and *bglC*) were multiply deleted in order to construct Δ*bglA*/*bglB* and Δ*bglA*/*bglB*/*bglC* mutants, respectively.

### Growth Properties of the β-Glucosidase Deletion Mutants

The growth properties of the wild type strain and the deletion mutants in liquid culture with glucose, cellobiose, and Avicel cellulose as the sole carbon source were tested. In glucose medium, the growth rates and final cell densities of all the deletion mutants were similar to that of the wild type strain (**Supplementary Figure S2A**). In cellobiose and Avicel cellulose medium, Δ*bglB* could reach the same cell density as the wild type strain but it had a longer lag phase. Δ*bglA*/*bglB* could not grow either in cellobiose or cellulose mediums, but the other deletion mutants grew as well as the wild type strain (**Supplementary Figures S2B,C**). The phenotypic properties of Δ*bglA*/*bglB*/*bglC* were similar to those of Δ*bglA*/*bglB* in all our experiments, indicating that Δ*bglC* did not play an obvious role in cellobiose and cellulose degradation. So the results of Δ*bglA*/*bglB*/*bglC* are not shown in our following experiments.

### β-Glucosidase and Endo-Glucanase Activity Determination of the β-Glucosidase Deletion Mutants

**FIGURE 2:**
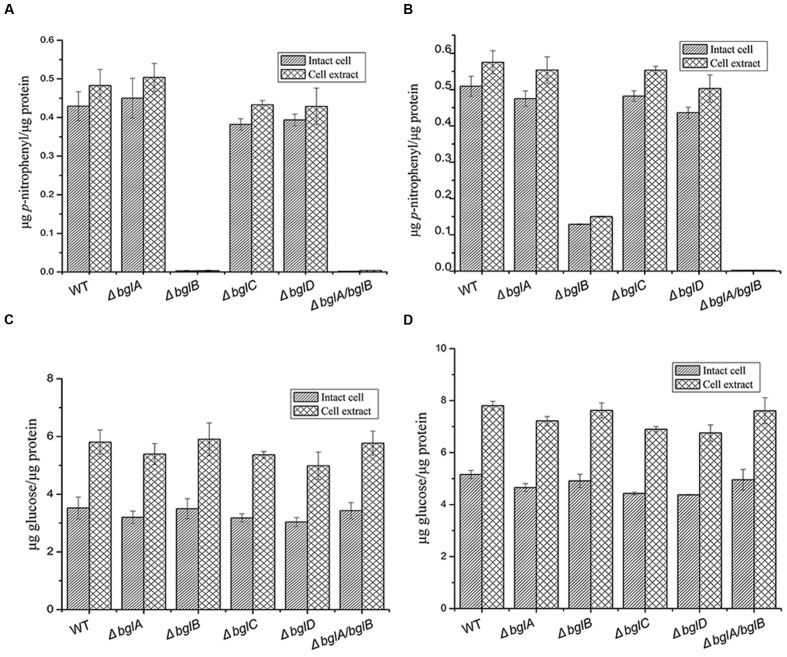
**β-Glucosidase and endo-glucanase activities of the wild type strain and β-glucosidase deletion mutants.** **(A)** β-Glucosidase activities of cells cultured in glucose. **(B)** β-Glucosidase activities of cells cultured in cellulose. **(C)** Endo-glucanase activities of cells cultured in glucose. **(D)** Endo-glucanase activities of cells cultured in cellulose. β-Glucosidase activity was determined using *p*NPG as the substrate, and endo-glucanase activity was determined using CMC-Na as the substrate. Values are the mean of three biological replicates. Error bars are the standard deviations from these replicates. Error bars indicate standard errors. Because the Δ*bglA*/*bglB* mutant could not grow in cellulose 0.05% glucose was added into the medium to ensure the growth of Δ*bglA*/*bglB* **(B,D)**, the wild type strain and other single deletion mutants were grown with cellulose as sole carbon source.

In order to investigate the function of β-glucosidases in cellulose degradation, β-glucosidase and endo-glucanase activities of the wild type strain and the deletion mutants were analyzed (**Figure 2**). In glucose culture, Δ*bglB* and Δ*bglA*/*bglB* completely lost β-glucosidase activity, while all the other single deletion mutants had similar β-glucosidases activities as the wild type strain. In cellulose culture, Δ*bglB* only kept about 20% of the wild type β-glucosidase activity while Δ*bglA*/*bglB* lost all the β-glucosidase activity. All the results indicated that BglB was the major β-glucosidase of *C. hutchinsonii*. BglA could be induced by cellulose and complement part of the β-glucosidase activity of Δ*bglB*. BglA together with BglB were the dominant β-glucosidase proteins of *C. hutchinsonii*. Endo-glucanase activities of the wild type strain and the β-glucosidase deletion mutants were roughly the same both for intact cells and total cell extract, indicating that expressions of the endo-glucanases were unaffected by the disruption of β-glucosidases.

### Cellobiose Utilization of the β-Glucosidase Deletion Mutants

**FIGURE 3:**
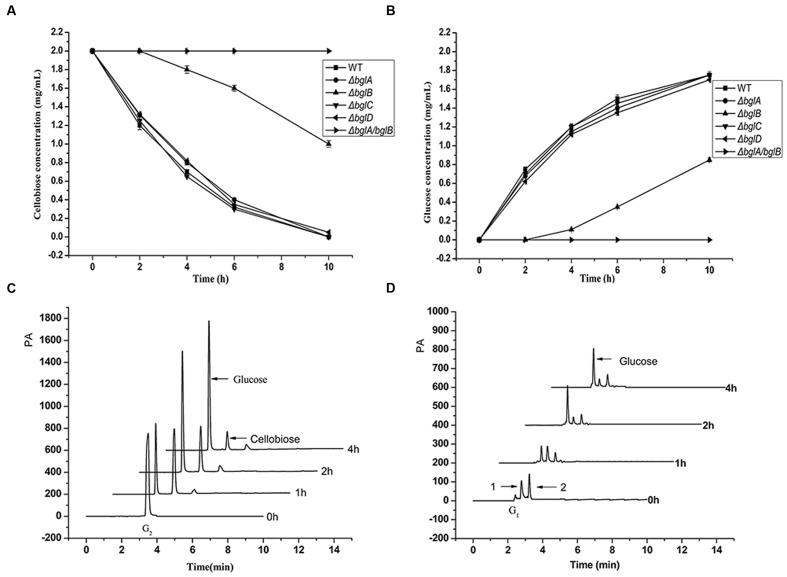
**Cellobiose utilization.** **(A,B)** Cellobiose degradation by the wild type strain and the β-glucosidase deletion mutants in Na_2_HPO_4_–KH_2_PO_4_ buffer (pH 6.8). **(A)** The remaining cellobiose and **(B)** the generated glucose in the medium. Cells (50 μg of protein per milliliter) were incubated with 0.2% (wt/vol) cellobiose in Na_2_HPO_4_–KH_2_PO_4_ buffer, in which condition the cell concentration kept stable. Values are the mean of three biological replicates. Error bars are the standard deviations from these replicates. **(C,D)** Cellobiose utilization by the wild type strain in Stanier medium. **(C)** Cellobiose degradation products in the medium supernatant and **(D)** the accumulated cello-oligosaccharides in the cells. Cells (100 μg of protein per milliliter) were incubated with 0.2% (wt/vol) cellobiose in Stanier medium. There were two small peaks (peak 1 and peak 2) between the sites of the glucose peak and cellobiose peak in the cell sample. PA, peak altitude.

To compare the cellobiose hydrolytic abilities of the wild type strain and the β-glucosidase deletion mutants, cells were incubated with 2.0 mg/mL cellobiose in Na_2_HPO_4_–KH_2_PO_4_ buffer (pH 6.8), and the degradation products were tested at different time intervals. As shown in **Figures 3A,B**, all the single deletion mutants except Δ*bglB* showed a similar cellobiose degradation rate as the wild type strain. They could degrade all the cellobiose into glucose in 10 h and a concentration of 1.8 mg/mL glucose accumulated in the medium. However, Δ*bglB* started to degrade cellobiose after a delay of 2 h and the degradation rate was much slower than that of the wild type strain. After 10 h, it could degrade only about half of the total cellobiose. But Δ*bglA*/*bglB* completely lost the ability to hydrolyze cellobiose. These results confirmed that BglA and BglB were the two essential β-glucosidases for cellobiose degradation.

In order to investigate how cellobiose was utilized by *C. hutchinsonii*, wild type strain cells were incubated with 2.0 mg/mL cellobiose in Stanier medium and the degradation products both in the medium supernatant and in the cells were detected by ion chromatography. As shown in the **Figure 3C**, almost all of the cellobiose disappeared and a large amount of glucose (about 1.45 mg/mL) accumulated in the medium within 4 h incubation, while there was almost no cellobiose and only a little glucose accumulated in the cells (**Figure 3D**). According to the result, we could draw a conclusion that *C. hutchinsonii* could degrade cellobiose and generate glucose in the medium rapidly. There were two small peaks (peak 1 and peak 2) between the glucose peak and the cellobiose peak in the intracellular sample. The compositions of the two peaks are still unknown, and we speculated they might be the metabolites of glucose in the cells.

### Cello-oligosaccharide Utilization of the β-Glucosidase Deletion Mutants

**FIGURE 4:**
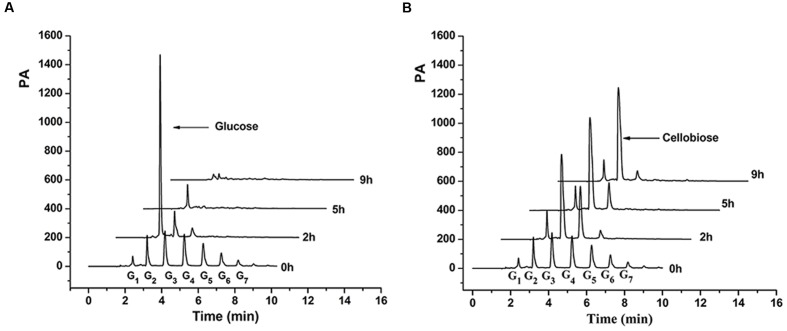
**Cello-oligosaccharides degradation products of wild type strain** **(A)** and Δ*bglA*/*bglB* **(B)** in the medium. G_1_, G_2_, G_3_, G_4_, G_5_, G_6_, and G_7_ stand for the glucose and other cello-oligosaccharides.

The utilization process of the cello-oligosaccharide mixture by the wild type strain and Δ*bglA*/*bglB* were further tested. As shown in **Figure 4**, long chain cello-oligosaccharide (G_3_–G_7_) quickly decreased along with an obvious accumulation of glucose in the medium of the wild type strain within 2 h (**Figure 4A**). Subsequently, the accumulated glucose was completely utilized by the cells. The intracellular hydrolytic products of the wild type cells were also tested during the process, and only a small amount of glucose was detected (data not shown). For Δ*bglA*/*bglB* (**Figure 4B**), long chain cello-oligosaccharide (G_3_–G_7_) also quickly disappeared with an obvious accumulation of cellobiose in 9 h. Cellotriose accumulated in the medium within 2 h, and then gradually degraded with the main products of cellobiose. Since there was no β-glucosidase activity in the mutant, cellobiose could not be degraded. The result indicated that extracellular soluble cello-oligosaccharides (DP < 7) were firstly degraded into glucose by β-glucosidases and then be utilized by the cells.

### Cellulose Utilization of the β-Glucosidase Deletion Mutants

**FIGURE 5:**
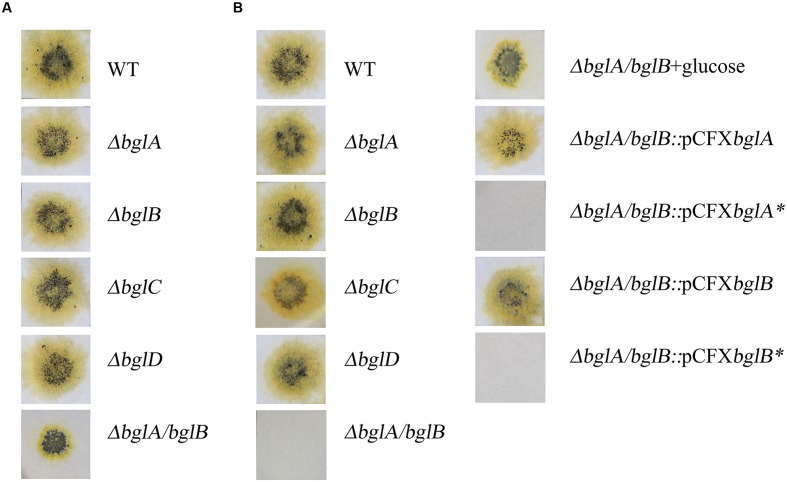
**Filter paper degradation on solid medium.** **(A)** Filter papers were laid on 10 g/liter Stanier agar. **(B)** Filter papers were laid on 10 g/liter Stanier phytagel with one exception: Δ*bglA*/*bglB*+glucose, filter paper was laid on Stanier phytagel, which was supplemented with 0.05% (wt/vol) glucose. Equal amounts of cells of the wild type strain and deletion mutants were spotted on filter paper and incubated at 30°C and recorded at 15 days. All measurements were performed in triplicate. Asterisk was used to mark the site mutational complemented plasmids and strains.

The mutants were examined for the ability to digest and grow on cellulose. All the single deletion mutants retained the ability to digest and grow on filter paper as the sole source of carbon and energy on Stanier agar plates (**Figure 5A**). Δ*bglA*/*bglB* could also digest filter paper but with a smaller degradation area. This was inconsistent with the result that Δ*bglA*/*bglB* could not utilize Avicel in liquid culture. The main difference of the two kinds of mediums was that there was agar in the solid medium.

**FIGURE 6:**
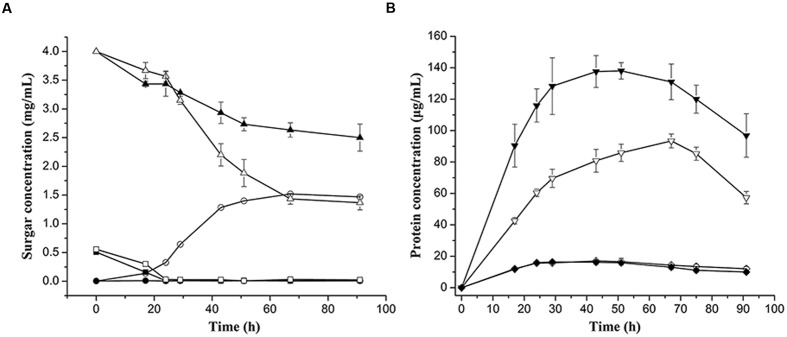
**Growth curves of the wild type strain and Δ*bglA*/*bglB* in 0.4 % (wt/vol) cellulose Stanier medium supplemented with 0.05% (wt/vol) glucose.** **(A)** Sugar concentration curves of glucose, cellobiose, and residual cellulose. The concentrations of glucose and cellobiose were measured by ion chromatography, and the concentrations of residual cellulose were determined by measuring the drying cellulose. Symbol indication: 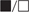, glucose; 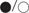 cellobiose; 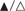, cellulose. **(B)** Protein concentrations of wild type strain and Δ*bglA*/*bglB*. Symbol indication: 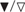, cells cultured in 0.4% (wt/vol) cellulose supplemented with 0.05 % (we/vol) glucose; 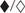, cells cultured in 0.05% (wt/vol) glucose. The closed and open symbols stand for samples of the wild type strain and Δ*bglA*/*bglB*, respectively. Values are the mean of three biological replicates. Error bars are the standard deviations from these replicates.

In order to make it clear whether agar could influence the growth of the mutants on cellulose, phytagel (a very pure agar substitute secreted by *Pseudomonas*) was used to replace agar in the solid medium. As shown in **Figure 5B**, Δ*bglA*/*bglB* could not digest filter paper on Stanier phytagel medium, while the wild type strain and single deletion mutants could still digest the filter paper. We deduced that the micro-nutrients in agar might facilitate cellulose utilization by Δ*bglA*/*bglB*. In order to detect the effect of the additional nutrients on cellulose utilization by Δ*bglA*/*bglB*, 0.05% (wt/vol) glucose was added into the cellulose medium both in the liquid medium and Stanier phytagel plates to test the growth of Δ*bglA*/*bglB*. As shown in **Figure 5B**, Δ*bglA*/*bglB* could digest filter paper with a smaller digestion area than the wild type strain on Stanier phytagel. Similarly, Δ*bglA*/*bglB* could partially degrade cellulose in the liquid cellulose medium with additional glucose (**Figure 6**).

However, Δ*bglA*/*bglB* could not degrade cellobiose even with additional glucose (**Supplementary Figure S3**). Moreover, the Δ*bglA*/*bglB* mutant could not grow in cellotriose, cellotetrose, or soluble cello-oligosaccharide mixed media even with additional glucose (data not shown). These results indicated that BglA and BglB were essential for cello-oligosaccharides utilization. But with additional glucose *C. hutchinsonii* could partially utilize cellulose even without BglA and BglB.

### Detection of the Cellulose Degradation Products

**FIGURE 7:**
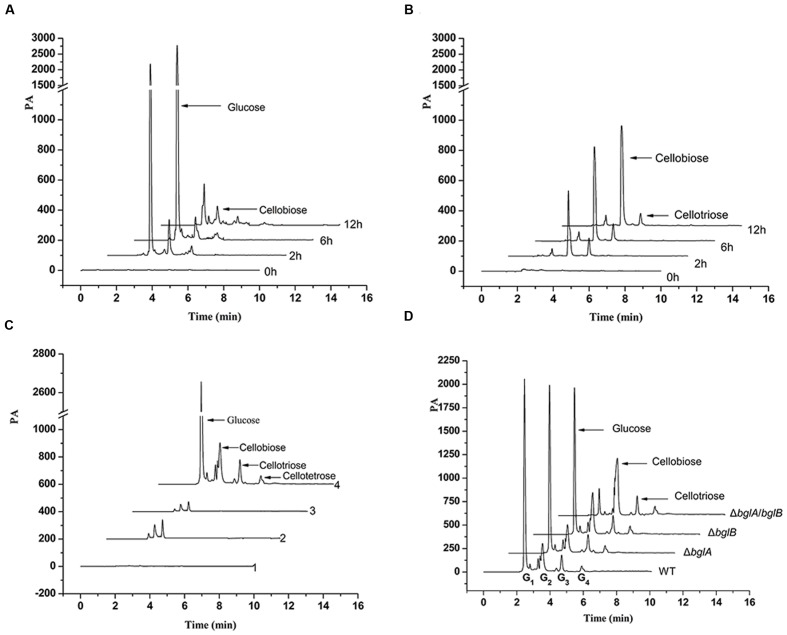
**Cellulose degradation products in the medium and in the cells.** **(A,B)** The cellulose degradation products of the wild type strain **(A)** and Δ*bglA*/*bglB* **(B)** in the medium. Cells were cultured in PY6 glucose medium to middle exponential phase and resuspended to a concentration of 100 μg of protein per milliliter in Stanier medium supplemented with 0.4% (wt/vol) cellulose. **(C)** The intracellular cello-oligosaccharides of the wild type strain under different culture conditions. Line 1, control sample, the residual metabolites in the Stanier medium after washing the cells for the third time; line 2, cells in glucose culture; line 3, free cells in cellulose culture; line 4, cellulose binding cells in cellulose culture. **(D)** The intracellular cello-oligosaccharides of cellulose binding cells in cellulose culture. All the samples were extracted from the same amount (100 μg of protein per milliliter) of the wild type strain and β-glucosidase deletion mutant.

In order to detect the cellulose degradation mechanism of *C. hutchinsonii* and the pathway that Δ*bglA*/*bglB* utilizes cellulose without β-glucosidase, the cellulose degradation products of the wild type strain and the Δ*bglA*/*bglB* mutant were tested. As shown in **Supplementary Figure S4**, there was glucose accumulated with a concentration as high as 0.003% (wt/vol) in the initial lag phase of the wild type strain when cultured in cellulose medium. After the lag phase, the extracellular glucose was almost undetectable. When wild type cells (100 μg of protein per milliliter) were incubated with 0.4% cellulose, large amounts of glucose accumulated in the medium between 2 and 6 h (**Figure 7A**). There was a concentration of about 0.004% glucose and 0.00023% cellobiose accumulated in the supernatant after incubation for 2 h. But after 12 h, only a small amount of glucose could be detected. A small amount of cellobiose and cellotriose could be detected throughout the test periods. But for Δ*bglA*/*bglB*, since it had no β-glucosidase activity, a large amount of cellobiose but almost no glucose was accumulated in the medium with time (**Figure 7B**).

The intracellular degradation products of the cells cultured in cellulose were also detected. When *C. hutchinsonii* was cultured in cellulose medium, we found that most of the cells were absorbed on the cellulose but there were also some free cells suspended in the medium (**Supplementary Figure S5**). The free cells and the cellulose-bound cells were separately collected and their intracellular cello-oligosaccharide products were tested. As shown in **Figure 7C**, there were significant amounts of glucose, cellobiose, cellotriose, and cellotetrose present in the cellulose-bound cells. Surprisingly, almost no glucose and cello-oligosaccharide was detected in the free cells, which was the same as the cells cultured in glucose medium.

The intracellular cello-oligosaccharides products of the β-glucosidase deletion mutants cultured in cellulose were also tested. As shown in **Figure 7D**, large amounts of glucose were detected in wild type strain and single β-glucosidase deletion mutants, but only a small amount of glucose was detected in Δ*bglA*/*bglB*. However, a large amount of cellobiose was accumulated in Δ*bglA*/*bglB*, and the cellobiose present in Δ*bglB* was more than that present in the wild type strain and Δ*bglA*. This result indicated that BglA and BglB played an important role in periplasmic cellobiose degradation.

### Complementation of Δ*bglA*/*bglB*

In order to verify that the defects in cellobiose and cellulose degradation of Δ*bglA*/*bglB* were caused by the deficiency of β-glucosidase activities, complementations of Δ*bglA*/*bglB* were carried out with plasmids carrying native *bglA*, *bglB*, and active site mutations *bglA*^∗^, *bglB*^∗^. Strains complemented with native *bglA* and *bglB* could both restore the ability to degrade cellulose (**Figure 5B**) and cellobiose (data not shown). However, the strains complemented with *bglA*^∗^ and *bglB*^∗^ could not restore the defects (**Figure 5B**). These results proved that the defects in cellobiose and cellulose degradation of the mutant are caused by the inactivation of β-glucosidase activities.

## Discussion

Cellobiose is one of the most important intermediate products of cellulose degradation. Different cellulolytic microorganisms utilize cellobiose in different pathways (Alexander, 1968; Zhang and Lynd, 2004; Zhang et al., 2011). In fungi and aerobic bacteria, cellobiose and other cello-oligosaccharides could be hydrolyzed by extracellular β-glucosidase, or by intracellular β-glucosidase after cellobiose is transported into the cell by cellodextrin transporters (Galazka et al., 2010). In anaerobic cellulolytic bacteria, cellulose is digested into cello-oligosaccharides by cellulosomes (Bayer and Lamed, 1992). The cellobiose and other cello-oligosaccharides are transported into the cell and they are digested by periplasmic β-glucosidases or phosphorylases (Strobel et al., 1995; Zhang and Lynd, 2004; Nataf et al., 2009). Cellobiose phosphorylases are thought to function in energy conservation to minimize ATP consumption during fermentative metabolism under stressful conditions, such as an anaerobic environment (Zhang and Lynd, 2004). In this study, we found that BglB was a constitutive expression protein and had a major role in the degradation of cellobiose, while BglA which was induced by cellobiose could partially make up for the deletion of *bglB*. All the four predicted β-glucosidases had been heterologously expressed in our lab, and the results showed that BglA and BglB were typical β-glucosidases, BglC had very low β-glucosidase activity but high transglycosylase activity, BglD had no cellulase or β-glucosidase activity (unpublished data). According to all the above results, we concluded that BglA and BglB were the crucial β-glucosidases for cellobiose degradation in *C. hutchinsonii*. We also found that BglA and BglB were mainly located in the periplasmic space (**Figures 1B,C**) and could degrade cellobiose and other cello-oligosaccharides generated by cellulases into glucose in the periplasmic space. Exogenous cellobiose and other cello-oligosaccharides could also be rapidly degraded into glucose by *C. hutchinsonii* cells (**Figures 3A–C** and **Figure 4A**).

The Δ*bglA*/*bglB* mutant lost the ability to grow in cellulose, and there was almost no glucose accumulated in the medium (**Figure 7B**). However, a small amount of added glucose could partially restore the mutant’s ability to utilize cellulose. For the wild type strain, a small amount of glucose appeared in the initial stage of growth in the cellulose medium (**Supplementary Figure S4**). All of results implied that glucose was essential for initiating cellulose degradation.

Direct contact between the cells and insoluble cellulose was supposed to be necessary for cellulose degradation by *C. hutchinsonii* (Walker and Warren, 1938; Stanier, 1942). In this work, we found there were both adherent cells and free cells when *C. hutchinsonii* was cultured in cellulose medium (**Supplementary Figure S5**). Since *C. hutchinsonii* could not secret free cellulases into the medium, the free cells could not degrade cellulose. The result that almost no cello-oligosaccharide was detected inside the free cells also supported this (**Figure 7C**). In the study, apparent glucose and cello-oligosaccharides could be detected in the medium when cells were incubated with cellulose. The study of the distribution of cell-contact cellulases showed that the enzyme activity of the intact cells, which represented the enzyme activity on the cell surface, possessed 60–64% of the total endo-glucanase activity. We deemed that the non-adherent cells in the medium might be fed by the cellulolytic products which were produced on the cell surface or leaked from the periplasmic space of the adherent cells. Considering that the free cells are more conducive to spread and set on a new substrate in the environment, feeding of the non-adherent cells by the adherent cells might be beneficial for the *C. hutchinsonii* community to explore new territory.

Our results showed that Δ*bgla*/*bglb* had no β-glucosidase activity (**Figure 2**) and it could not degradate cellobiose (**Figure 3A**), but Δ*bglA*/*bglB* could partially degrade cellulose with the addition of a small amount of exogenous glucose. This indicates that *C. hutchinsonii* had the ability to utilize cellulose without β-glucosidases. Quantitative analysis showed that when Δ*bglA*/*bglB* was grown in cellulose medium with 0.05% glucose, about 2.7 mg/mL of the cellulose was hydrolyzed with 1.5 mg/mL of cellobiose accumulated in the medium (**Figure 6A**). In addition, there was some cellobiose accumulated in the cell (**Figure 7D**). These results indicated that more than 55% of the degraded cellulose was converted into cellobiose by Δ*bglA*/*bglB*, and the other part of the degraded cellulose might be converted into glucose to supply the growth of the mutant. Our previous work reported that endo-glucanases from *C. hutchinsonii* such as CHU_1280 and CHU_2103 could both hydrolyze RAC to produce cellobiose and glucose (Zhang et al., 2014, 2015), which also supported our speculation that *C. hutchinsonii* could convert cellulose into glucose without β-glucosidases. This study increased our understanding of the complicated cellulolytic system of *C. hutchinsonii*.

## Author Contributions

XB, XW, and XL conceived and designed the experiments. XB, XW, SW, XJ, and WZ performed the experiments. XB, XW, SW, XJ, and WZ analyzed the data. XB, XW, SW, XJ, WZ, and XL wrote the paper. ZG, WZ, and XL revised the manuscript. All authors read and approved the final manuscript.

## Conflict of Interest Statement

The authors declare that the research was conducted in the absence of any commercial or financial relationships that could be construed as a potential conflict of interest.
