# Supplementary material for: Functional Studies of β-Glucosidases of Cytophaga hutchinsonii and Their Effects on Cellulose Degradation
Source: Front Microbiol. 2017 Feb 2;8:140. doi: 10.3389/fmicb.2017.00140 (PMC5288383; doi:10.3389/fmicb.2017.00140)
Supplement: Supplementary file 1 [file Data_Sheet_1.DOC]

**Functional studies of beta-glucosidases of *Cytophaga hutchinsonii* and their effects on cellulose degradation**

Xinfeng Bai^a^, Xifeng Wang^a^, Sen Wang^a^, Xiaofei Ji^b^, Zhiwei Guan^a^, Weican Zhang and Xuemei Lu^a*^

State Key Laboratory of Microbial Technology, School of Life Science, Shandong University, Jinan, China^a^; Department of Pathogenic Biology, Binzhou Medical University, Shandong Province, Yantai, China^b^.

Running Title: β-glucosidase function and cellulose degradation

*Address correspondence to Xuemei Lu, [luxuemei@sdu.edu.cn](mailto:luxuemei@sdu.edu.cn)

**Table S1** Primers used in this study.

| Primer | Sequence^a^ |
| --- | --- |
| Primers used for RT-PCR assays | |
| 16*SrRNA*-1 AAGGGTGAAACTCAAAGGA  16*SrRNA*-2 CTCGCTGGCAACTAAAGAT | |
| RT- *bglA* -1 | GCAATGAATCGCTGTATCCTG |
| RT- *bglA* -2 | TGATTACTCCAGGCTGTTCG |
| RT- *bglB* -1 | CAACTCCGGTGAGATCAATG |
| RT- *bglB* -2 | ATTGTAGGGCTGATGTTGTG |
| RT- *bglC* -1 | AGAACTACACGGAGCCTGGTC |
| RT- *bglC* -2 | CAAGTACATCAGCAGCACG |
| RT- *bglD* -1 | GAAGAAAGTCCGAACCACATC |
| RT- *bglD* -2 | AAGAATACGGCCCGGTTGAG |
|  |  |
| Primers used for construction of deletion plasmids | |
| *bglA-*H1F | ATCTAAGCATGCGGATAATTATACGCTCAC |
| *bglA-*H1R | GACGCTATCTAGATTTTAGACGTCAGGTCAC |
| *bglA-*H2F | GCACTTGGTACCGCTGTAATAATGTGCTAC |
| *bglA-*H2R | ATCGTCGGATCCAGTGTTCAAAAATTCTTC |
| *bglB-*H1F | GTGGTGTCTAGATTCACTGTGGTTCCTGCCAT |
| *bglB-*H1R | ACGTTGGAGCTCGATTTCTTTGTCTTTGTCTG |
| *bglB-*H2F | TCCTTCGGTACCTGGATGCTGGTCATACACCT |
| *bglB-*H2R | GTCATGGGATCCGCTACCTGTTTATACAGGAA |
| *bglC-*H1F | ATGAAATCTAGAACCTGGTAGGAGTGAATG |
| *bglC-*H1R | CGAATGGAGCTCATTTAATGTATACGCATG |
| *bglC-*H2F | TATGTAGGTACCATCAACGGGAAAGCAGAC |
| *bglC-*H2R | GGACTTGGATCCTTAGCTCAGCAGTTATTC |
| *bglD-*H1F | AATTACGCATGCAGACATTTCATGGGTTGC |
| *bglD-*H1R | CTCGTCGAGCTCCATTTTTTTAACCAAGGC |
| *bglD-*H2F | GTTAAAGGGTACCTGATTGTAACACTGTCGG |
| *bglD-*H2R | AAGTTTGGATCCCAAAAAGATCACGACGAC |
|  |  |
| Primers used for testing gene deletion mutants | |
| *bglA-*F | AAGATAATACCAGCAAGACAC |
| *bglA-*R | AGAACCCACTACACCAACATC |
| *bglB-*F | GTTCTGTTTCGTGTTCCTCC |
| *bglB-*R | TGCTTTGTCCTGAATCTGTG |
| *bglC*-F | GCAGATAACGGTCAAACAACA |
| *bglC-*R | TTAACGTATGTGTGCCCTCAT |
| *bglD-*F | ACAAACGCAAACCTGATAAACC |
| *bglD*-R | TGGTTCGGACTTTCTTCCAC |
|  |  |
| Primers used for construction of complementation plasmids | |
| C*bglA-*H1F | ATGTAGGAGCTCATCGTTATTTGCTATTGC |
| C*bglA*-H1R | CAAACGGATCACATCTTCCCAAGCCGATACAACCATTCCGG |
| C*bglA-*H2F | CCGGAATGGTTGTATCGGCTTGGGAAGATGTGATCCGTTTG |
| C*bglA-*H2R | TTTAAAGTCGACTCCCTTCTGCCTATTCAG |
| C*bglB-*H1F | TTTGTAGAGCTCGATAACCAGCCGTATTCG |
| C*bglB-*H1R | CGGTTGATATCTTCCCAGGCTGTTACAATCAAACCTTTG |
| C*bglB-*H2F | CAAAGGTTTGATTGTAACAGCCTGGGAAGATATCAACCG |
| C*bglB-*H2R | ATACAAGTCGACCCTCCCGCAAATTGCAGG |

^a^ Restriction sites on the primers are underlined

**
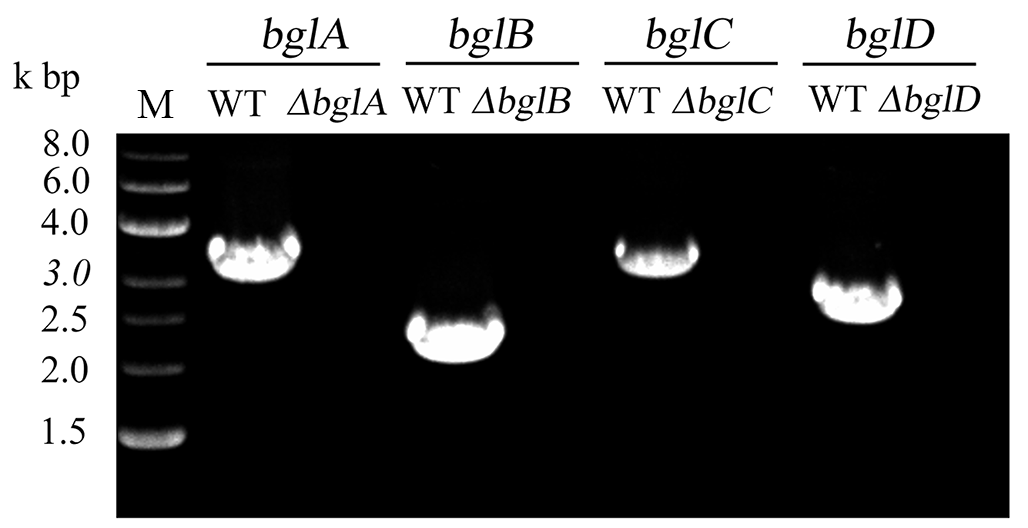
**

**Fig. S1.** PCR confirmation of deletion of the regions spanning *bglA*, *bglB*, *bglC* and *bglD* in wild type strain and the corresponding single deletion mutants. WT, wild-type strain; M, DNA marker. Prime pairs *bglA-*F/*bglA-*R, *bglB-*F/*bglB-*R, *bglC-*F/*bglC-*R and *bglD-*F/*bglD-*R were used to confirm the deletion of regions spanning *bglA*, *bglB*, *bglC* and *bglD*, respectively.


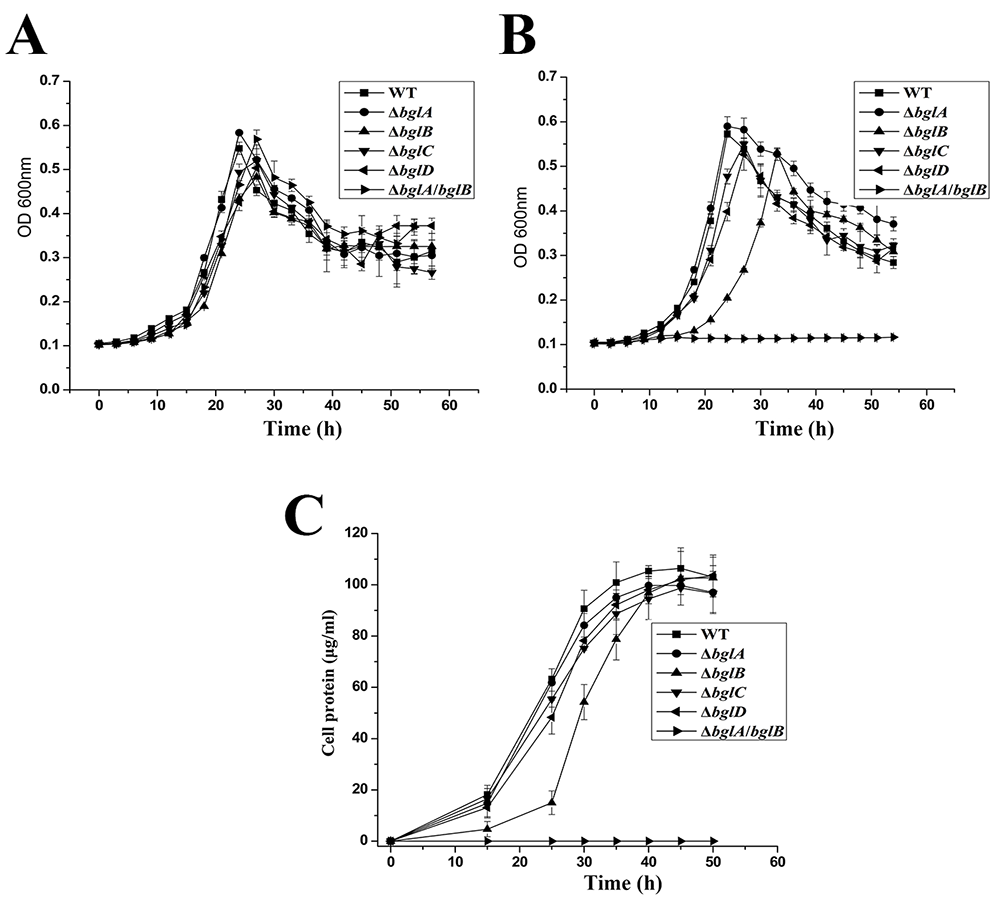


**Fig S2.** Growth curves of the wild type strain and β-glucosidase deletion mutants in Stanier medium with different carbon sources. (A) 0.2% (wt/vol) glucose. (B) 0.2% (wt/vol) cellobiose. (C) 0.4% (we/vol) Avicel. Values are the mean of three biological replicates. Error bars are the SD from these replicates.


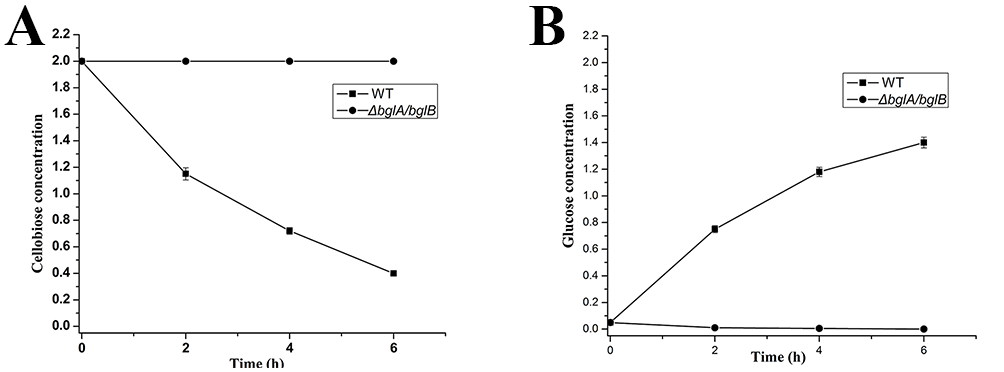


**Fig** S**3.** **Cellobiose utilization.** Cellobiose degradation by the wild type strain and the Δ*bglA*/*bglB* deletion mutant in Na_2_HPO_4_-KH_2_PO_4_ buffer (pH 6.8). (A) the remaining cellobiose and (B) the generated glucose in the medium. Cells (50 μg of protein per milliliter) were incubated with 0.2% (wt/vol) cellobiose and 0.05% glucose in Na_2_HPO_4_-KH_2_PO_4_ buffer, in which condition the cell concentration kept stable. Values are the mean of three biological replicates. Error bars are the standard deviations from these replicates.


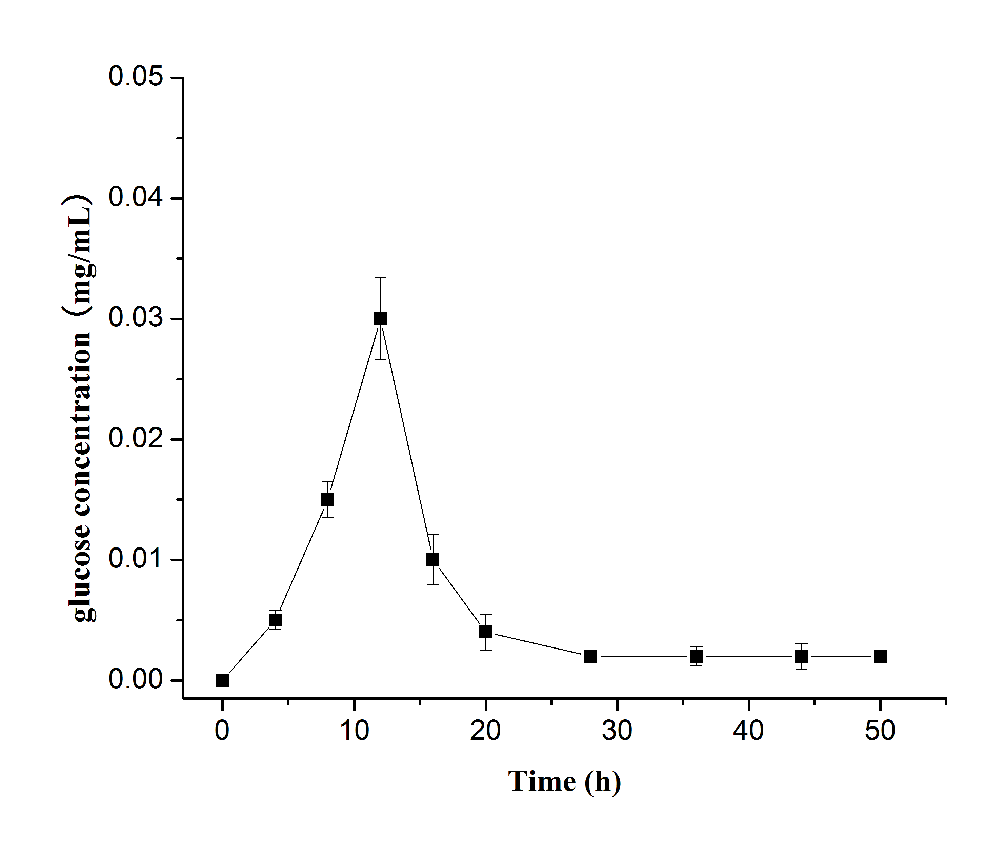


**Fig. S4.** The glucose concentration of wild type strain in the cellulose medium. 3% (vol/vol) of wild type strain cells were inoculated into Stanier medium supplemented with o.4% (wt/vol) cellulose. The glucose concentration in the supernatant was detected by ion chromatography. Values are the mean of three biological replicates. Error bars are the SD from these replicates.


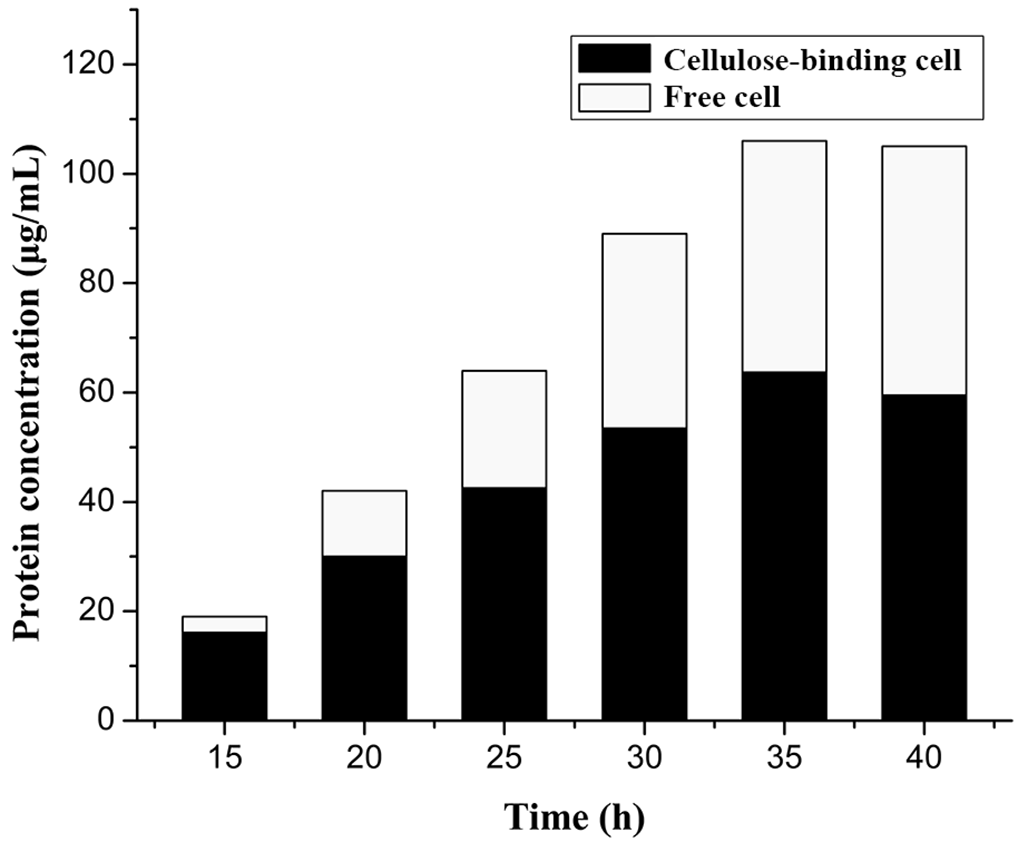


**Fig. S5.** The protein concentration of free cells and cellulose-binding cells in cellulose culture. 3% (vol/vol) of wild type strain cells were inoculated into100ml of Stanier medium supplemented with o.4% (wt/vol) cellulose. The free cells and the cellulose-bound cells were separated and collected respectively as described in the material and method. Then the total cell protein concentration was quantified.
